# Clinical features and outcomes associated with macrolide resistance - associated mutations in pediatric *Mycoplasma pneumoniae* pneumonia

**DOI:** 10.1186/s13052-026-02310-w

**Published:** 2026-07-16

**Authors:** Yilan Huang, Zili Zhang, Ming Liang, Jiaxing Xu, Chengyu Lu, Jili Yuan

**Affiliations:** 1 State Key Laboratory of Respiratory Disease, Guangdong Key Laboratory of Vascular Disease, National Clinical Research Center for Respiratory Disease, National Center for Respiratory Medicine, Guangzhou Institute of Respiratory Health, The First Affiliated Hospital of Guangzhou Medical University, Guangzhou, Guangdong China; 2 Department of Pediatrics, The First Affiliated Hospital of Guangzhou Medical University, Guangzhou, Guangdong China; 3 Guangzhou Medical University, Guangzhou, Guangdong China

**Keywords:** *Mycoplasma pneumoniae* pneumonia, Azithromycin, Macrolide resistance-associated mutations, Severe Mycoplasma pneumoniae pneumonia, Children

## Abstract

**Objective:**

To investigate the clinical characteristics of macrolide resistance - associated mutations in hospitalized children with *Mycoplasma pneumoniae* pneumonia (MPP) and to evaluate their associations with severe *Mycoplasma pneumoniae* pneumonia (SMPP) and prolonged cough.

**Methods:**

A total of 1,094 hospitalized children with MPP were retrospectively enrolled. Among them, 1,069 underwent A2063G/A2064G mutation testing, whereas 25 were not tested. Patients were classified into mutation-positive, mutation-negative, and untested groups according to mutation testing results. Clinical features, laboratory parameters, treatment profiles, and outcomes were compared among groups. Logistic regression and receiver operating characteristic (ROC) curve analyses were performed to identify risk factors for SMPP and prolonged cough.

**Results:**

The proportion of SMPP was higher in the mutation-positive group than in the mutation-negative group. In addition, patients in the mutation-positive group exhibited a more pronounced inflammatory response and greater immune imbalance, as reflected by increased neutrophil percentage and D - dimer levels, along with decreased CD4 and CD8 counts. Univariate analysis showed that A2063G/A2064G mutation status was associated with both SMPP and prolonged cough. However, multivariable analysis identified fibrinogen and AST as independent factors associated with SMPP, while prolonged cough was additionally associated with the duration of azithromycin and clarithromycin therapy, as well as CD8 levels.

**Conclusion:**

Children with macrolide resistance-associated mutations exhibited a higher inflammatory burden and a higher unadjusted proportion of SMPP. However, after adjustment, A2063G/A2064G mutation status was not independently associated with SMPP or prolonged cough. Clinical management should integrate inflammatory markers, oxygenation status, and clinical presentation for individualized monitoring and treatment.

**Supplementary Information:**

The online version contains supplementary material available at https://doi.org/10.1186/s13052-026-02310-w.

## Introduction

*Mycoplasma pneumoniae* (MP) is a major cause of community - acquired pneumonia (CAP) in children, particularly in preschool - and school - aged populations, accounting for approximately 10% − 40% of pediatric CAP cases [1–4]. Although MPP is usually self - limiting, some children may develop SMPP [5, 6], characterized by worsening symptoms, prolonged fever, progressive radiographic lesions, or extrapulmonary complications [7–10]. Even with conventional macrolide therapy, these cases often require further evaluation and comprehensive management. When SMPP is accompanied by cardiovascular, digestive, or neurological involvement, elevated white blood cell counts, neutrophil proportions, and C - reactive protein (CRP) levels are often observed, indicating more severe disease and possible impairment of pulmonary function and quality of life [8, 11].

Macrolides are the first - line treatment for pediatric MPP and inhibit MP protein synthesis by targeting domain V of 23 S rRNA [12]. However, macrolide resistance has increased rapidly in recent years [13, 14]. The A2063G and A2064G mutations are the main molecular basis of resistance [15–17], with detection rates reported to exceed 90% in pediatric MPP cases in China [18, 19]. Although resistance may prolong disease course and increase the risk of complications, in vitro resistance does not necessarily indicate clinical failure of azithromycin, and some children may still benefit from treatment [18, 20–23].

In addition to antimicrobial resistance, disease severity in MP infection is closely associated with host immune responses. Elevated CRP, LDH, D - dimer, and neutrophil proportions have been associated with prolonged disease [2, 18]. Excessive immune activation may aggravate lung injury through the Toll - like receptor 2 (TLR2) - myeloid differentiation primary response 88 (MyD88) - nuclear factor κB (NF - κB) pathway and inflammatory cytokine release [1, 19]. Moreover, long non - coding RNA metastasis - associated lung adenocarcinoma transcript 1 (MALAT1), age, immune status, and co - infection may also affect disease severity and prognosis [2, 19].

The treatment of macrolide-resistant MP infection in children remains challenging, as alternative agents such as tetracyclines and fluoroquinolones are limited by age and safety concerns [24]. Clinically, early identification of high - risk children and comprehensive evaluation of inflammatory and immune status are essential to optimize azithromycin use and guide adjunctive immunomodulatory therapy [18, 25]. However, although macrolide resistance-associated mutations are common in pediatric MPP, it remains unclear whether these mutations independently increase the risks of SMPP and prolonged cough after adjustment for these factors.

The novelty of this study lies in moving beyond simple comparisons of clinical phenotypes between mutation-positive and mutation-negative patients by further evaluating whether macrolide resistance-associated mutations are independent risk factors for adverse clinical outcomes. This may help determine whether detection of these mutations alone is sufficient to justify discontinuing azithromycin and switching to alternative antibiotics, thereby providing evidence for clinical management.

## Methods

### Study design and population

This retrospective observational cohort study was conducted in the Department of Pediatrics at the First Affiliated Hospital of Guangzhou Medical University. Hospitalized children diagnosed with MPP between January 2023 and December 2024 were retrospectively enrolled and stratified according to A2063G / A2064G mutation status. The inclusion criteria were as follows: (i) hospitalized children and (ii) diagnosis of pediatric MPP [6]. The exclusion criteria were: (i) resolution of fever and complete disappearance of cough before admission; (ii) incomplete clinical data that could not meet the requirements for analysis; (iii) presence of hematologic malignancies; (iv) chronic granulomatous disease; (v) purulent meningitis; (vi) immunodeficiency disorders; and (vii) no use of azithromycin during treatment. The study was approved by the Institutional Review Board (GZMC 2009-08-1336) and adhered to the Declaration of Helsinki as previously described. The requirement for informed consent was waived due to the retrospective design. No human or animal experiments were conducted. All procedures were performed in accordance with the Declaration of Helsinki (1964) and its subsequent amendments or comparable ethical standards.

### Data collection and definitions

Clinical data were retrospectively collected, including demographic characteristics, hospitalization information, underlying diseases and comorbidities, laboratory findings, chest imaging results, treatment regimens during hospitalization, duration of symptoms, diagnostic results, and clinical outcomes. The diagnostic criteria for MPP and SMPP were based on the Chinese guidelines for the diagnosis and treatment of pediatric MPP [6]. MPP was defined as: (i) positive MP - DNA or RNA detection; and / or (ii) a single serum MP antibody titer ≥ 1:160 (particle agglutination method), or a ≥ 4 - fold increase or a decrease to one - quarter in paired serum antibody titers during the disease course. SMPP was defined as confirmed MPP meeting any of the following criteria: (i) persistent high fever, defined as a body temperature ≥ 39 °C for ≥ 5 days, or fever lasting ≥ 7 days with no decrease or a further increase in peak body temperature; (ii) the presence of any respiratory symptom, including wheezing, shortness of breath, dyspnea, chest pain, or hemoptysis; (iii) extrapulmonary organ involvement that did not meet the criteria for critical illness; (iv) peripheral oxygen saturation ≤ 0.93 at rest while breathing room air at sea level; (v) radiographic findings meeting any of the following conditions: (1) high-density consolidation involving ≥ 2/3 of a single lung lobe, or high-density consolidation involving two or more lung lobes regardless of the extent of involvement; (2) diffuse unilateral or bilateral bronchiolitis, with or without bronchitis, including bronchial wall thickening, peribronchial exudation, and mucus plug formation leading to atelectasis; (vi) progressive worsening of clinical symptoms accompanied by radiographic progression of lesions by more than 50% within 24–48 h; or (vii) marked elevation of at least one inflammatory or tissue injury marker, including CRP, LDH, or D-dimer. As there is no universally accepted pathogen-specific cutoff for prolonged cough in pediatric Mycoplasma pneumoniae infection, prolonged cough was defined as a total cough duration exceeding 27 days, corresponding to the median value of this cohort. This cutoff is close to 4 weeks and falls within the clinically recognized range of prolonged acute cough in children [26–28]. A structured follow - up protocol was implemented for all patients. A follow - up visit was scheduled at the respiratory outpatient clinic one week after discharge, during which a comprehensive evaluation based on symptoms and signs was performed. Chest computed tomography or radiography was conducted when persistent cough, wheezing, or abnormal oxygenation was present. Routine imaging follow - up was not performed in asymptomatic patients.

### Detection of macrolide resistance - associated mutations

Among the 1,094 included patients, 1,069 underwent A2063G/A2064G mutation testing using bronchoalveolar lavage fluid (BALF) samples, whereas 25 patients were not tested. In patients who underwent BALF examination, A2063G/A2064G mutations were detected using nested polymerase chain reaction combined with capillary electrophoresis and single-strand conformation polymorphism analysis (nPCR-CE-SSCP), according to previously reported methods [29].

### Clinical outcome assessment

The primary outcomes of this study were the occurrence of SMPP and prolonged cough.

### Statistical analysis

All statistical analyses were performed using Python 3.11 and R Studio (version 2026.01.1 + 403). Continuous variables were tested for normality, and most were non - normally distributed; therefore, they were expressed as median and interquartile range (IQR). Comparisons between two groups were performed using the Mann - Whitney U test, while comparisons among three or more groups were conducted using the Kruskal - Wallis H test. Categorical variables were presented as counts and percentages and analyzed using Pearson’s χ² test or Fisher’s exact test, as appropriate. A two - sided P value < 0.05 was considered statistically significant.

Univariate logistic regression analysis was performed for all candidate variables. Variables with *P* < 0.05 in univariate analysis were included in multivariate logistic regression models to estimate adjusted odds ratios (ORs) and 95% confidence intervals (95% CIs) [18, 20]. Multicollinearity was assessed prior to model construction, and variables with significant collinearity were selected based on clinical relevance and statistical performance. Receiver operating characteristic (ROC) curve analysis was further used to evaluate the predictive performance of relevant indicators for SMPP and prolonged cough [30, 31].

## Results

### Study population

**Fig. 1:**
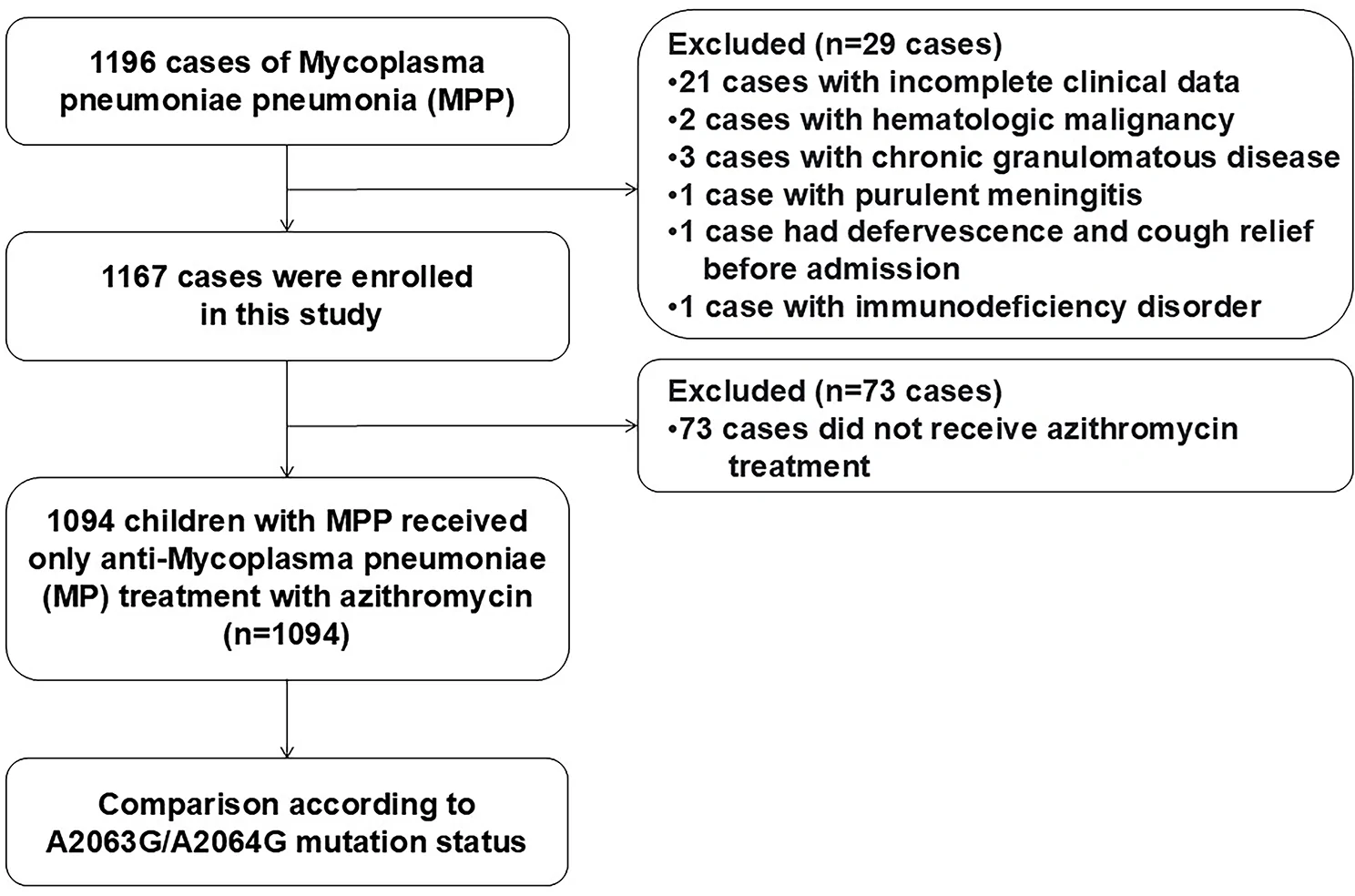
Study flowchart

During the study period from January 2023 to December 2024, a total of 1,196 pediatric cases initially met the inclusion criteria. Of these, 29 patients were excluded, including those with resolution of fever and complete disappearance of cough before admission (*n* = 1), incomplete clinical data (*n* = 21), hematologic malignancies (*n* = 2), chronic granulomatous disease (*n* = 3), purulent meningitis (*n* = 1), and immunodeficiency disorders (*n* = 1). Among the remaining 1,167 patients, 73 who did not receive azithromycin during treatment were further excluded. Finally, 1,094 eligible patients were included in the analysis (Fig. 1).

All included patients received azithromycin therapy. Among them, 958 patients were positive for A2063G/A2064G mutations (mutation-positive group), 111 were negative for A2063G/A2064G mutations (mutation-negative group), and 25 were not tested for these mutations (untested group).

### Clinical characteristics of 1,094 children with MPP

A total of 1,094 children with MPP were included in the analysis. The median age was 6.00 years, and 571 patients (52.2%) were male. Bacterial and viral co-infections were identified in 321 (29.3%) and 244 (22.3%) patients, respectively. The median total duration of fever and cough was 7.00 and 27.00 days, respectively. Most pulmonary lesions were completely resolved among patients who completed follow-up imaging. Detailed demographic, laboratory, treatment, clinical course, and imaging findings are summarized in Table 1 and Supplementary Table 1.

**Table 1:** Clinical characteristics of 1,094 children with MPP

Category / Variable	Overall Results
**Demographic Data**	
Age (years), median (IQR)	6.00 (4.00–8.00)
Male, n (%)	571 (52.2%)
**Infection-Related Indicators**	
Bacterial co-infection, n (%)	321 (29.3%)
Viral co-infection, n (%)	244 (22.3%)
**Laboratory Findings**	
PaO₂, median (IQR)	85.55 (75.41–96.20)
White blood cell count (×10⁹/L), median (IQR)	7.54 (6.05–9.62)
Neutrophils (%), median (IQR)	60.10 (50.00-68.53)
Lymphocytes (%), median (IQR)	29.30 (22.30-38.32)
CRP (mg/L), median (IQR)	1.30 (0.54–2.38)
LDH(U/L), median (IQR)	314.00 (272.00-363.55)
Fibrinogen (g/L), median (IQR)	4.29 (3.72–4.80)
D-dimer, median (IQR)	733.50 (487.00-1088.50)
CD3, median (IQR)	1356.00 (997.00-1941.00)
CD4, median (IQR)	734.00 (526.00-1089.00)
CD8, median (IQR)	459.00 (320.00-665.00)
**Treatment**	
Methylprednisolone use, n (%)	317 (29.0%)
Duration of azithromycin use (days), median (IQR)	6.00 (4.00–6.00)
**Clinical Course**	
Duration of fever before treatment (days), median (IQR)	6.00 (4.00–7.00)
Total duration of fever (days), median (IQR)	7.00 (5.00–9.00)
Total duration of cough (days), median (IQR)	27.00 (22.00–33.00)
**Imaging Findings (Initial Imaging)**	
Atelectasis, n (%)	46/801 (5.74%)

IQR, interquartile range; PaO₂, arterial partial pressure of oxygen; CRP, C-reactive protein; LDH, lactate dehydrogenase; CD, cluster of differentiation; BALF, bronchoalveolar lavage fluid. Percentages for initial imaging findings were calculated using the number of patients who underwent initial imaging examination as the denominator. Percentages for follow-up imaging outcomes were calculated using the number of patients who completed follow-up imaging as the denominator. Percentages for other categorical variables were calculated using the total number of patients in each group as the denominator

### Comparison of clinical characteristics among three groups

Compared with the mutation-negative group, the mutation-positive group had a higher proportion of SMPP and showed greater inflammatory burden and immune imbalance, as reflected by elevated inflammatory markers and lower lymphocyte subset counts. The mutation-positive group also had a longer fever course, indicated by a lower proportion of patients with fever duration ≤ 3 days, and more frequent use of clarithromycin, methylprednisolone, and doxycycline. The untested group did not show an obviously poorer short-term clinical course than the tested groups, but this finding should be interpreted cautiously because of the small sample size. Detailed between-group comparisons, including laboratory findings, treatment profiles, clinical course, and imaging outcomes, are presented in Table 2 and Supplementary Table 2.

**Table 2:** Comparison of clinical characteristics and treatment outcomes among children with MPP Stratified by A2063G/A2064G mutation status

Category / Variable	Mutation-Positive Group	Mutation-Negative Group	Untested Group	*P* value
**Demographic Data**				
Age (years), median (IQR)	6.00 (4.00–8.00)	5.00 (3.00–8.00)	6.00 (3.00–9.00)	0.1737
Male, n (%)	496 (51.77%)	62 (55.86%)	13 (52.00%)	0.7173
**Infection-Related Indicators**				
Bacterial co-infection, n (%)	258 (26.93%)	55 (49.55%)	8 (32.00%)	< 0.0001***
Viral co-infection, n (%)	191 (19.94%)	49 (44.14%)	4 (16.00%)	< 0.0001***
**Laboratory Findings**				
PaO₂, median (IQR)	85.30 (75.00-95.78)	88.20 (78.70–99.10)	90.70 (85.00-94.60)	0.3870
White blood cell count (×10⁹/L), median (IQR)	7.47 (6.07–9.51)	8.55 (6.13-11.00)	7.46 (5.84–8.76)	0.0294*
Neutrophils (%), median (IQR)	60.90 (51.70–69.00)	48.60 (40.10–63.10)	52.60 (47.30–63.70)	< 0.0001***
Lymphocytes (%), median (IQR)	28.25 (21.80-36.82)	37.10 (27.15–48.10)	37.00 (24.50–39.40)	< 0.0001***
CRP (mg/L), median (IQR)	1.35 (0.60–2.38)	0.85 (0.11–2.19)	1.05 (0.70–2.66)	0.0035**
LDH (U/L), median (IQR)	315.00 (275.00-364.50)	293.00 (249.40-349.75)	324.50 (287.05–347.00)	0.0499*
Fibrinogen (g/L), median (IQR)	4.31 (3.80–4.81)	3.74 (3.01–4.63)	4.16 (3.11–4.80)	< 0.0001***
D-dimer, median (IQR)	763.00 (528.50-1107.50)	464.00 (275.50–711.00)	691.50 (467.50-1168.50)	< 0.0001***
CD3, median (IQR)	1310.50 (985.25-1869.50)	2005.00 (1224.75-2577.75)	1580.00 (1037.00-1934.50)	< 0.0001***
CD4, median (IQR)	707.00 (513.00-1010.25)	1098.50 (658.75-1427.25)	764.00 (615.00-1090.50)	< 0.0001***
CD8, median (IQR)	434.50 (314.75–647.00)	608.00 (432.50-863.50)	483.00 (326.00-642.50)	< 0.0001***
Neutrophils in BALF (%), median (IQR)	93.50 (88.00-96.25)	88.50 (71.62–94.56)	82.38 (65.06–89.31)	< 0.0001***
Macrophages in BALF (%), median (IQR)	3.12 (1.25-8.00)	7.25 (2.25–23.75)	12.25 (6.31–29.81)	< 0.0001***
**Treatment**				
Clarithromycin use, n (%)	520 (54.28%)	34 (30.63%)	11 (44.00%)	< 0.0001***
Doxycycline use, n (%)	238 (24.84%)	14 (12.61%)	5 (20.00%)	0.0146*
Methylprednisolone use, n (%)	295 (30.79%)	18 (16.22%)	4 (16.00%)	0.0021**
Duration of methylprednisolone use (days), median (IQR)	2.00 (2.00–3.00)	3.00 (2.00-5.50)	1.00 (1.00–2.00)	0.0381*
**Clinical Course**				
Total duration of fever (days), median (IQR)	7.00 (5.00–9.00)	5.00 (0.50-8.00)	7.00 (5.00–7.00)	< 0.0001***
Total duration of cough (days), median (IQR)	27.00 (22.00–33.00)	27.00 (21.00-35.25)	24.00 (19.50–26.00)	0.0390*
SMPP, n(%)	679(70.9%)	64(57.7%)	13 (52.0%)	0.0030**
Fever duration ≤ 3 days, n (%)	160 (16.70%)	48 (43.20%)	5 (20.00%)	< 0.0001***
**Imaging Findings (Initial Imaging)**				
Atelectasis, n (%)	32/694 (4.61%)	14/85 (16.47%)	0/22 (0.00%)	0.0003***

IQR, interquartile range; PaO₂, arterial partial pressure of oxygen; CRP, C-reactive protein; LDH, lactate dehydrogenase; CD, cluster of differentiation; BALF, bronchoalveolar lavage fluid; SMPP, severe mycoplasma pneumoniae pneumonia. Percentages for initial imaging findings were calculated using the number of patients who underwent initial imaging examination as the denominator. Percentages for follow-up imaging outcomes were calculated using the number of patients who completed follow-up imaging as the denominator. Percentages for other categorical variables were calculated using the total number of patients in each group as the denominator. * *P* < 0.05, ** *P* < 0.01, *** *P* < 0.001

### Risk factor analysis for SMPP

**Fig. 2:**
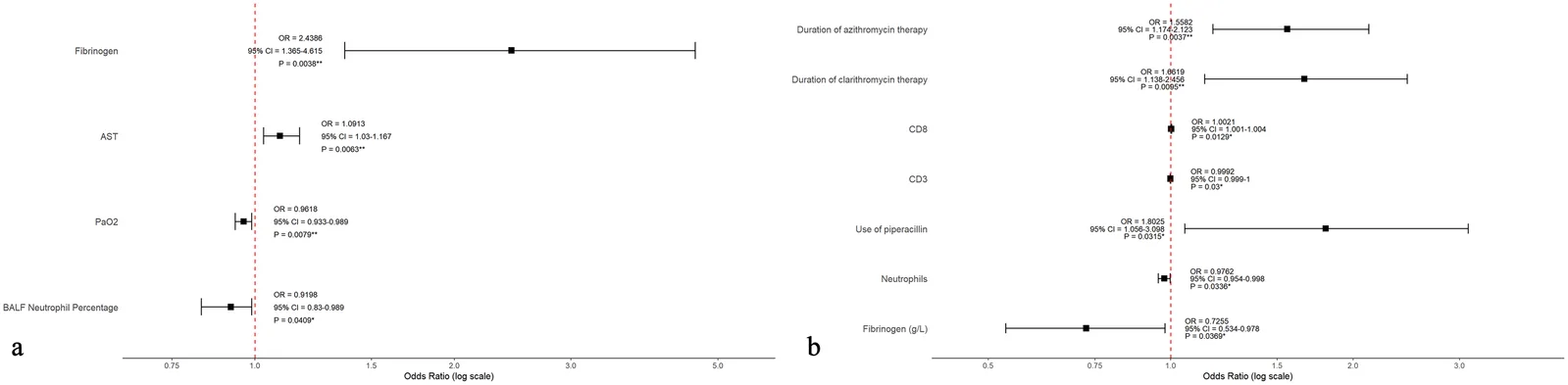
Forest plots of SMPP and prolonged cough. The forest plots show the odds ratios (ORs) and 95% confidence intervals (CIs) for (**a**) SMPP and (**b**) prolonged cough, derived from multivariable logistic regression analysis.* P < 0.05, ** P < 0.01, *** P < 0.001

In the analysis of SMPP, several inflammatory, immune-related, oxygenation, and BALF indicators were significant in univariate analysis. In the multivariable model, fibrinogen and AST remained independent risk factors, whereas PaO₂ and BALF neutrophil percentage were independently associated with a lower risk of SMPP. ROC analysis showed that PaO₂ had the best discriminatory performance among the evaluated indicators. Detailed regression and ROC results are shown in Tables 3 and 4; Fig. 2.

**Table 3:** Logistic regression analysis of risk factors for SMPP in children

Variable	Univariate Logistic Regression			Variables	Multivariable Logistic Regression		
	OR	95%CI	*P* value		OR	95%CI	*P* value
PaO₂	0.9681	0.9553–0.9801	< 0.001***	Fibrinogen	2.4386	1.3655–4.6150	0.0038**
Lymphocyte	0.9764	0.9667–0.9860	< 0.001***	AST	1.0913	1.0299–1.1671	0.0063**
Neutrophil	1.0222	1.0129–1.0316	< 0.001***	PaO₂	0.9618	0.9333–0.9889	0.0079**
Fibrinogen	1.3547	1.1688–1.5729	< 0.001***	BALF Neutrophil Percentage	0.9198	0.8301–0.9888	0.0409*
Methylprednisolone	1.6899	1.2573–2.2907	0.0006***				
BALF Macrophage	0.9809	0.9680–0.9934	0.0031**				
BALF Neutrophil Percentage	1.0164	1.0046–1.0287	0.0064**				
CD3	0.9998	0.9996-1.0000	0.0105*				
AST	1.0140	1.0038–1.0262	0.0150*				
ALT	1.0136	1.0021–1.0282	0.0427*				
Mutation-positive group	2.2465	0.9988–5.0126	0.0465*				

IQR, interquartile range; CI, confidence interval; OR, odds ratio; PaO₂, arterial partial pressure of oxygen; BALF, bronchoalveolar lavage fluid; CD, cluster of differentiation; AST, aspartate transaminase; ALT, alanine transaminase. * *P* < 0.05, ** *P* < 0.01, *** *P* < 0.001

**Table 4:** ROC curve analysis of key indicators for predicting SMPP

Risk Factor	Cutoff Value	Sensitivity	Specificity	AUC (95% CI)	*P* value
Fibrinogen	4.14	0.6237	0.5104	0.5779 (0.541–0.6148)	< 0.0001***
AST	34.65	0.4960	0.6339	0.5739 (0.538–0.6099)	< 0.0001***
PaO₂	85.80	0.5881	0.7167	0.6898 (0.6363–0.7433)	< 0.0001***
BALF Neutrophil Percentage	90.38	0.6627	0.4925	0.5865 (0.5285–0.6445)	0.0034**

CI, confidence interval; AUC, area under the receiver operating characteristic curve; PaO₂, arterial partial pressure of oxygen; BALF, bronchoalveolar lavage fluid. * *P* < 0.05, ** *P* < 0.01, *** *P* < 0.001

### Risk factor analysis for prolonged cough

For prolonged cough, univariate analysis identified multiple inflammatory, immune-related, treatment-related, and clinical variables as significant factors. In the multivariable model, the duration of azithromycin therapy, duration of clarithromycin therapy, CD8 level, and piperacillin use were associated with prolonged cough, whereas CD3 level, neutrophil proportion, and fibrinogen were associated with a lower risk. ROC analysis indicated limited predictive performance of individual indicators. Detailed results are shown in Tables 5 and 6, Supplementary Tables 3, and Fig. 2.

**Table 5:** Logistic regression analysis of risk factors for prolonged cough in children with MPP

Variables	Univariate Logistic Regression			Variables	Multivariable Logistic Regression		
	OR	95%CI	*P* value		OR	95%CI	*P* value
Neutrophils	0.9911	0.9827–0.9995	0.0382*	Duration of azithromycin therapy	1.5582	1.1742–2.1226	0.0037**
Untested group	4.3033	1.5188–15.4782	0.0116*	Duration of clarithromycin therapy	1.6619	1.1379–2.4561	0.0095**
Mutation-positive group	4.2756	1.6130-14.7397	0.0082**	CD8	1.0021	1.0006–1.0039	0.0129*
CD3	1.0003	1.0001–1.0004	0.0012**	CD3	0.9992	0.9985–0.9999	0.0300*
Duration of azithromycin therapy	1.3619	1.1627–1.6027	0.0002***	Use of piperacillin	1.8025	1.0556–3.0983	0.0315*
Duration of azithromycin use (days)	1.1163	1.0563–1.1811	0.0001***	Neutrophils	0.9762	0.9543–0.9979	0.0336*
Use of piperacillin	1.7246	1.3117–2.2707	< 0.0001***	Fibrinogen (g/L)	0.7255	0.5342–0.978	0.0369*
CD8	1.0010	1.0005–1.0014	< 0.0001***				
Fibrinogen (g/L)	0.7444	0.6469–0.8549	< 0.0001***				

CI, confidence interval; OR, odds ratio; CRP, C-reactive protein; CD, cluster of differentiation. * *P* < 0.05, ** *P* < 0.01, *** *P* < 0.001

**Table 6:** ROC curve analysis of key indicators for predicting prolonged cough

Risk Factor	Cutoff Value	Sensitivity	Specificity	AUC (95% CI)	*P* value
Duration of azithromycin therapy	2.5000	0.1313	0.8982	0.5097 (0.4744–0.5449)	0.5974
Duration of clarithromycin therapy	1.5000	0.4004	0.7083	0.5555 (0.5119–0.5992)	0.0176*
CD8	442.5000	0.5262	0.6465	0.5941 (0.5499–0.6382)	< 0.0001***
CD3	1320.5000	0.5212	0.6414	0.5875 (0.5421–0.6329)	0.0002***
Piperacillin use	0.5000	0.2619	0.7690	0.5154 (0.4864–0.5445)	0.3078
Neutrophils	54.6500	0.6863	0.4727	0.5973 (0.5583–0.6363)	< 0.0001***
Fibrinogen (g/L)	3.8250	0.7528	0.4234	0.6009 (0.5615–0.6404)	< 0.0001***

CI, confidence interval; AUC, area under the receiver operating characteristic curve; CRP, C-reactive protein; CD, cluster of differentiation. * *P* < 0.05, ** *P* < 0.01, *** *P* < 0.001

### Baseline characteristics and clinical outcomes of mutation-positive children according to doxycycline use

Among mutation-positive patients, those who received doxycycline after macrolide therapy had more severe baseline disease than those treated with macrolides alone, including older age, longer fever duration, higher inflammatory burden, and lower CD4 and CD8 levels. Because baseline characteristics were not balanced between groups, this analysis should be interpreted as a descriptive subgroup comparison rather than evidence of doxycycline inefficacy. Multivariable analysis showed that doxycycline use was not independently associated with SMPP, and no significant difference in the incidence of prolonged cough was observed between the two treatment groups. Detailed results are summarized in Table 7 and Supplementary Tables 4 and 5.

**Table 7:** Baseline characteristics and clinical outcomes of mutation-positive children according to doxycycline use

Category / Variable	Macrolide Only (*n* = 720)	Macrolide Followed by Doxycycline (*n* = 238)	*P* value
**Baseline Characteristics**			
Age	6 (3–7)	8 (5.25-9)	< 0.0001***
Duration of fever	7 (5–9)	8 (6–9)	0.0018**
**Disease Severity**			
PaO₂	85.2 (75-95.1)	85.4 (75-98.5)	0.8340
Oxygen saturation	93 (90.5–95)	90 (89–92)	0.0841
**Laboratory Findings**			
Neutrophils	59.55 (50.48–68.03)	64.65 (56.6–72)	< 0.0001***
CRP (mg/L)	1.26 (0.49–2.3)	1.59 (0.82–2.52)	0.0010**
D-dimer	726 (491-1080.5)	852 (626.5-1157.25)	0.0011**
CD4	748 (552-1078.75)	605.5 (460.5-871.5)	< 0.0001***
CD8	460 (325–676)	396 (289.25-566.25)	0.0004***
**Infection-Related Indicators**			
Bacterial infection	200 (27.8%)	58 (24.4%)	0.3134
Viral infection	150 (20.8%)	41 (17.2%)	0.2614
**Treatment**			
Duration of azithromycin therapy	2 (1–2)	1 (1–1)	< 0.0001***
Use of methylprednisolone	193 (26.8%)	102 (42.9%)	< 0.0001***

IQR, interquartile range; PaO₂, arterial partial pressure of oxygen; CRP, C-reactive protein; CD, cluster of differentiation. * *P* < 0.05, ** *P* < 0.01, *** *P* < 0.001

## Discussion

This retrospective cohort study of hospitalized children with MPP demonstrated that patients harboring A2063G/A2064G mutations exhibited a higher inflammatory burden and a greater unadjusted proportion of SMPP. However, after adjustment for inflammatory, immune, oxygenation, and treatment-related variables, mutation status was not independently associated with SMPP or prolonged cough. The main contribution of this study is that it shows that macrolide resistance-associated mutations should not be considered the sole determinant of adverse clinical outcomes, and that clinical interpretation of mutation status should be integrated with inflammatory burden, immune status, oxygenation, and treatment-related factors.

The mutation-positive group showed more pronounced abnormalities in inflammatory and immune parameters, consistent with previous reports [32] indicating that macrolide-resistant MPP is often accompanied by stronger inflammatory responses. However, mutation status was not retained as an independent risk factor in multivariable models, suggesting that the apparent association with adverse outcomes may be partially explained by enhanced host inflammatory responses and immune dysregulation rather than by the resistance mutation per se [6, 33–35]. Previous studies have also shown that a subset of children harboring A2063G/A2064G mutations can still achieve short-term clinical responses to azithromycin: one study reported that 36 of 155 patients (23.23%) defervesced within 3 days of treatment [18], while another study reported a mean time to defervescence of 4.6 days [4]. Consistently, in our cohort, 160 of 958 (16.70%) mutation-positive patients achieved defervescence within 3 days, indicating that the presence of resistance-associated mutations does not necessarily predict poor short-term clinical response to azithromycin.

Compared with mutation status, inflammatory markers, indicators of tissue or organ injury, oxygenation status, and immune parameters showed stronger associations with SMPP and prolonged cough. Fibrinogen and AST were independent risk factors for SMPP, whereas PaO₂ was a protective factor. For prolonged cough, treatment duration and immune indicators were retained in the multivariable regression model. These findings suggest that disease progression in pediatric MPP is driven by multiple pathophysiological pathways and cannot be explained solely by A2063G/A2064G mutation status [18, 36].

Subgroup analysis of resistance-associated mutations further supports an individualized management approach. Children who received doxycycline after macrolide therapy had more severe baseline disease, but doxycycline use was not independently associated with SMPP, and the incidence of prolonged cough did not differ significantly between groups. This does not indicate inefficacy of doxycycline but highlights that clinical decision-making should be guided by overall disease severity and individual patient risk. Mechanistically, our findings are biologically plausible. MP infection can activate the TLR2-MyD88-NF-κB innate immune pathway [1], and MP - derived community - acquired respiratory distress syndrome (CARDS) toxin may further trigger inflammasome activation and cytokine release [37], with excessive host inflammatory responses contributing to lung injury and prolonged symptoms [38]. These mechanisms also support the observation that inflammatory and immune status may be more closely associated with disease progression than the presence of resistance mutations.

Based on these findings, the study has important clinical implications: detection of A2063G/A2064G mutations alone should not be used as the sole criterion for discontinuing azithromycin [18, 20–22]. The broadly comparable short-term clinical course in the small untested group also suggests that routine mutation testing may not be required for every patient, particularly in selected low-risk cases. Treatment of macrolide-resistant MP infection in children remains challenging because alternative agents, such as tetracyclines and fluoroquinolones, are limited by age and safety considerations [24]. Clinical decision-making should therefore integrate overall disease severity, treatment response, patient age, drug safety, and inflammatory burden, with dynamic assessment of the potential need for adjunctive corticosteroids or intravenous immunoglobulin in children with more severe disease or at higher risk of progression [3, 39, 40].

This study has several limitations. First, it is a retrospective observational study in which treatment regimens were determined by attending clinicians according to clinical judgment rather than by randomization, which may introduce confounding by indication; consequently, the analysis of doxycycline use should be interpreted as an association rather than as evidence of treatment efficacy. Second, although multiple inflammatory, immune, and clinical variables were adjusted for, residual confounding cannot be fully excluded. Third, the study was conducted at a single center and included only hospitalized children, limiting the generalizability of the findings to milder outpatient cases. Fourth, not all patients had both baseline and follow-up imaging available, which may have introduced baseline assessment bias and follow-up bias. Fifth, 25 patients did not undergo mutation testing, and although they were included as an untested group, the small sample size limits the interpretability of comparisons; future studies with larger untested cohorts could further validate these findings. Sixth, because there is currently no universally accepted clinical threshold for defining prolonged cough in pediatric MP infection, the 27-day cutoff used in this study was data-driven and based on the median total cough duration in this cohort. If a standardized guideline-based cutoff becomes available in the future, the outcomes could be reanalyzed using that definition. Finally, prospective multicenter cohort studies with standardized resistance testing, predefined cough outcome thresholds, and more rigorous methods to control confounding, such as propensity score matching or randomized controlled trials, are warranted to validate these findings and establish optimal management strategies for children with resistance-associated MPP.

## Conclusion

This study showed that A2063G/A2064G mutation status was not an independent risk factor for SMPP. In contrast, indicators of inflammation, immune status, oxygenation, and liver function were more strongly associated with SMPP and prolonged cough. These findings suggest that mutation testing alone should not guide treatment decisions, and that clinical management should be based on a comprehensive assessment of inflammatory status, oxygenation, and disease progression.

## Supplementary Information

Below is the link to the electronic supplementary material.

Supplementary Material 1

## Data Availability

The datasets used and/or analysed during the current study are available from the corresponding author on reasonable request.

## Abbreviations

MPP: Mycoplasma pneumoniae pneumonia
SMPP: Severe Mycoplasma pneumoniae pneumonia
ROC: Receiver operating characteristic
CRP: C-reactive protein
CAP: Community-acquired pneumonia
TLR2: Toll-like receptor 2
MyD88: Myeloid differentiation primary response 88
NF-κB: Nuclear factor κB
MALAT1: Metastasis-associated lung adenocarcinoma transcript 1
IRB: Institutional Review Board
BALF: Bronchoalveolar lavage fluid
nPCR-CE-SSCP: nested polymerase chain reaction combined with capillary electrophoresis and single-strand conformation polymorphism analysis
IQR: Interquartile range
OR: Odds ratio
CI: Confidence interval
PaO₂: Arterial partial pressure of oxygen
MUMPP: Macrolide-unresponsive Mycoplasma pneumoniae pneumonia
CARDS: Community-acquired respiratory distress syndrome
NLRP3: NLR family pyrin domain containing 3
TNF-α: Tumor necrosis factor-α
IL: Interleukin
