# Supplementary material for: Clinical features and outcomes associated with macrolide resistance - associated mutations in pediatric Mycoplasma pneumoniae pneumonia
Source: Ital J Pediatr. 2026 Jul 16;52:151. doi: 10.1186/s13052-026-02310-w (PMC13670534; doi:10.1186/s13052-026-02310-w)
Supplement: Supplementary file 1 [file 13052_2026_2310_MOESM1_ESM.docx]

Supplementary Table 1

| Category / Variable | Overall Results |
| --- | --- |
| **Underlying Diseases and Comorbidities** |  |
| Asthma, n (%) | 72 (6.6%) |
| Sinusitis, n (%) | 132 (12.1%) |
| Rhinitis, n (%) | 263 (24.0%) |
| BO, n (%) | 22 (2.0%) |
| **Laboratory Findings** |  |
| AST (U/L), median (IQR) | 33.50 (28.30-40.30) |
| ALT (U/L), median (IQR) | 13.30 (10.80-17.30) |
| Oxygen saturation (%), median (IQR) | 92.00 (90.00-95.00) |
| MP-IgM, median (IQR) | 7.77 (1.32-32.24) |
| Neutrophils in BALF (%), median (IQR) | 93.00 (86.94-96.00) |
| Macrophages in BALF (%), median (IQR) | 3.50 (1.50-8.62) |
| **Treatment** |  |
| Cephalosporin use, n (%) | 676 (61.8%) |
| Piperacillin use, n (%) | 278 (25.4%) |
| Clarithromycin use, n (%) | 565 (51.6%) |
| Doxycycline use, n (%) | 257 (23.5%) |
| IVIG use, n (%) | 648 (59.2%) |
| Course of azithromycin therapy, median (IQR) | 2.00 (1.00-2.00) |
| **Clinical Course** |  |
| Duration of cough before treatment (days), median (IQR) | 7.00 (5.00-10.00) |
| **Imaging Findings (Initial Imaging)** |  |
| Patchy opacities, n (%) | 360/801 (44.94%) |
| Bronchiolitis, n (%) | 181/801 (22.60%) |
| Involvement of ≥2/3 of a single lobe, diffuse unilateral involvement, or bronchiolitis involving ≥4/5 of both lungs, n (%) | 84/801 (10.49%) |
| Pulmonary consolidation, n (%) | 607/801 (75.78%) |
| Pleural effusion, n (%) | 45/801 (5.62%) |
| Ground-glass opacities, n (%) | 151/801 (18.85%) |
| **Imaging Findings (Follow-up Imaging)** |  |
| Complete resolution of pneumonia, n (%) | 218/247 (88.26%) |
| Residual pulmonary consolidation, n (%) | 31/247 (12.55%) |
| Residual bronchiolitis obliterans, n (%) | 15/247 (6.07%) |

IQR, interquartile range; BO, bronchiolitis obliterans; AST, aspartate aminotransferase; ALT, alanine transaminase; BALF, bronchoalveolar lavage fluid; IVIG, intravenous immunoglobulin. Unless otherwise specified, percentages were calculated using the total number of included patients as the denominator. Percentages for imaging outcomes, if applicable, were calculated using the number of patients who completed the corresponding imaging follow-up as the denominator.

Supplementary Table 2

| Category / Variable | Mutation-Positive Group | Mutation-Negative Group | Untested Group | P value |
| --- | --- | --- | --- | --- |
| **Underlying Diseases and Comorbidities** |  |  |  |  |
| Asthma, n (%) | 56 (5.85%) | 16 (14.41%) | 0 (0.00%) | 0.0743 |
| **Laboratory Findings** |  |  |  |  |
| AST (U/L), median (IQR) | 33.60 (28.50-40.30) | 32.60 (26.40-40.80) | 35.10 (27.90-40.40) | 0.4989 |
| ALT (U/L), median (IQR) | 13.30 (10.80-17.20) | 13.50 (10.65-19.80) | 13.50 (11.20-15.10) | 0.7104 |
| Oxygen saturation (%), median (IQR) | 92.00 (90.00-95.00) | 90.50 (89.25-96.25) | - | 0.7938 |
| MP-IgM, median (IQR) | 8.32 (1.42-33.20) | 4.91 (0.98-22.33) | 1.94 (0.52-11.38) | 0.0407* |
| **Treatment** |  |  |  |  |
| Piperacillin use, n (%) | 233 (24.32%) | 42 (37.84%) | 3 (12.00%) | 0.0025** |
| **Clinical Course** |  |  |  |  |
| Duration of fever before treatment (days), median (IQR) | 6.00 (4.00-7.00) | 4.00 (0.00-6.00) | 6.00 (4.00-7.00) | <0.0001*** |
| Duration of cough before treatment (days), median (IQR) | 7.00 (5.00-10.00) | 9.00 (5.00-14.00) | 6.00 (4.00-8.00) | 0.0018** |
| **Imaging Findings (Follow-up Imaging)** |  |  |  |  |
| Complete resolution of pneumonia, n (%) | 194/211 (91.94%) | 20/31 (64.52%) | 4/5 (80.00%) | 0.0005** |
| Residual bronchiolitis obliterans, n (%) | 10/211 (4.74%) | 5/31 (16.13%) | 0/5 (0.00%) | 0.0714 |

IQR, interquartile range; AST, aspartate aminotransferase; ALT, alanine transaminase; BALF, bronchoalveolar lavage fluid. Percentages for initial imaging findings were calculated using the number of patients who underwent initial imaging examination as the denominator. Percentages for follow-up imaging outcomes were calculated using the number of patients who completed follow-up imaging as the denominator. Percentages for other categorical variables were calculated using the total number of patients in each group as the denominator. * P < 0.05, ** P < 0.01, *** P < 0.001.

Supplementary Table 3

| Variables | Univariate Logistic Regression | | |
| --- | --- | --- | --- |
|  | OR | 95%CI | P |
| MP-IgM | 1.0013 | 1.0003-1.0025 | 0.0205* |
| CD4 | 1.0003 | 1.0001-1.0006 | 0.0118* |
| Lymphocytes | 1.0121 | 1.0027-1.0216 | 0.0115* |
| Duration of clarithromycin therapy | 1.4418 | 1.0943-1.9074 | 0.0097** |
| White blood cell count | 1.0583 | 1.0240-1.0956 | 0.0010** |
| Viral co-infection | 1.7034 | 1.2798-2.2712 | 0.0003*** |
| Platelet count | 1.0021 | 1.0010-1.0032 | 0.0002*** |

CI, confidence interval; OR, odds ratio; CD, cluster of differentiation. * P < 0.05, ** P < 0.01, *** P < 0.001.

Supplementary Table 4

| Category / Variable | Macrolide Only (n = 720) | Macrolide Followed by Doxycycline (n = 238) | P value |
| --- | --- | --- | --- |
| **Laboratory Findings** |  |  |  |
| Lymphocytes | 29.7 (22.6-38.12) | 25.75 (19.8-32.52) | <0.0001*** |
| PCT | 0.09 (0.05-0.18) | 0.1 (0.05-0.18) | 0.3639 |
| LDH | 318 (277.1-367.7) | 297.5 (265.75-344) | 0.0043** |

IQR, interquartile range; PCT, procalcitonin; LDH, lactate dehydrogenase. * P < 0.05, ** P < 0.01, *** P < 0.001.

Supplementary Table 5

| Variables | Univariate Logistic Regression | | | Variables | Multivariable Logistic Regression | | |
| --- | --- | --- | --- | --- | --- | --- | --- |
|  | OR | 95%CI | P |  | OR | 95%CI | P |
| Use of methylprednisolone | 1.74 | 1.24-2.49 | 0.0016** | Use of methylprednisolone | 1.66 | 1.17-2.40 | 0.0052** |
| Macrophages in BALF (%) | 0.98 | 0.97-1.00 | 0.0083** |  |  |  |  |
| Neutrophils in BALF (%) | 1.02 | 1.00-1.03 | 0.0125* |  |  |  |  |
| ALT (U/L) | 1.02 | 1.00-1.04 | 0.0311* |  |  |  |  |
| Oxygen saturation | 0.64 | 0.29-0.94 | 0.0320* |  |  |  |  |

CI, confidence interval; OR, odds ratio; BALF, bronchoalveolar lavage fluid; ALT, alanine transaminase. * P < 0.05, ** P < 0.01, *** P < 0.001.
